# European dog owner perceptions of obesity and factors associated with human and canine obesity

**DOI:** 10.1038/s41598-018-31532-0

**Published:** 2018-09-06

**Authors:** Alberto Muñoz-Prieto, Liza Rosenbaum Nielsen, Roman Dąbrowski, Charlotte Reinhard Bjørnvad, Josefin Söder, Elsa Lamy, Ingrida Monkeviciene, Blanka Beer Ljubić, Iosif Vasiu, Sara Savic, Francesca Busato, Zeki Yilmaz, Antonio F. Bravo-Cantero, Malin Öhlund, Sónia Lucena, Rasa Zelvyte, Jasna Aladrović, Pia Lopez-Jornet, Marco Caldin, Catarina Lavrador, Birute Karveliene, Vladimir Mrljak, Jovita Mazeikiene, Asta Tvarijonaviciute

**Affiliations:** 1 Interlab-UMU, Regional Campus of International Excellence “Mare Nostrum”, University of Murcia, Ed.16, 4a planta, Espinardo, Murcia, 30100 Spain; 2 Department of Veterinary and Animal Sciences, Faculty of Health and Medical Sciences, University of Copenhagen, Grønnegårdsvej 8, 1870 Frederiksberg C, Denmark; 3 Department and Clinic of Animal Reproduction, Faculty of Veterinary Medicine, University of Life Sciences, Gleboka 30 St, 20-612 Lublin, Poland; 4 Department of Veterinary Clinical Sciences, Faculty of Health and Medical Sciences, University of Copenhagen, Dyrlægevej16, 1870 Frederiksberg C, Denmark; 5 Department of Anatomy, Physiology and Biochemistry, Swedish University of Agricultural Sciences, P.O. Box 7011, SE_750 07 Uppsala, Sweden; 6 Instituto de Ciências Agrárias e Ambientais Mediterrânicas (ICAAM), Escola de Ciências e Tecnologia, Universidade de Évora, Núcleo da Mitra, Apartado 94, 7006-554 Évora, Portugal; 7 Department of Anatomy and Physiology, Research Center of Digestive Physiology and Pathology, Veterinary Academy, Lithuanian University of Health Sciences, Tilzes 18, LT-47181 Kaunas, Lithuania; 8 Clinic for Internal Diseases, Faculty of Veterinary Medicine, Heinzelova 55, 10000 Zagreb, Croatia; 9 Small Animal Emergency Hospital, University of Agricultural Sciences and Veterinary Medicine Cluj-Napoca, Calea Mănăştur 3-5, Cluj-Napoca, 400372 Romania; 10 Scientific Veterinary Institute “Novi Sad”, Novi Sad, Serbia; 11 San Marco Veterinary Clinic, Padova, Italy; 12 Department of Internal Medicine, Faculty of Veterinary Medicine, Uludag University, 16059 Nilufer, Bursa Turkey; 13 Estadistica Murcia, 30565 Las Torres de Cotillas, Murcia Spain; 14 Department of Clinical Sciences, Swedish University of Agricultural Sciences, Box 7054, 750 07 Uppsala, Sweden; 15 Department of Physiology and Radiobiology, Faculty of Veterinary Medicine, Heinzelova 55, 10000 Zagreb, Croatia; 16 Department of Oral Medicine, Faculty of Medicine, University of Murcia, 30100 Espinardo, Spain; 17 Veterinary faculty, Dr. L Kriaučeliūnas Small Animal Clinic (teaching hospital), Veterinary Academy, Lithuanian University of Health Sciences, Tilzes 18, LT-47181 Kaunas, Lithuania; 18 InMedica Vilnius - Alfa Clinic, Baltrusaicio 3, 06120 Vilnius, Lithuania

## Abstract

Obesity is a common nutrition-related disorder leading to reduced life expectancy in both humans and dogs. With the aim of identifying new prevention and control options, the study objectives were (1) to investigate dog-owner perceptions about obesity in terms of themselves and their dogs, and (2) to identify factors associated with obesity and possible social, environmental and economic drivers for its development in dog owners and their pets. A cross-sectional questionnaire-based study was performed across multiple countries. The questionnaire focused on human and canine obesity, associated factors and potential drivers, and was distributed online and in the form of hard copies among dog owners in 11 European countries. In total, 3,185 responses from ten countries were included in multivariable analyses. Between 19.1% and 48.8% of the dog owners reported to be overweight/obese. Owner-reported overweight/obesity in dogs ranged from 6.0% to 31.3% based on body condition score charts, and 31.8% to 69.4% based on body fat index charts. Common factors associated with obesity in owners and their dogs were age, gender and owners’ attitudes to diet and physical activity. Dog owners who did not consider obesity to be a disease were more likely to have obese dogs.

## Introduction

Obesity is one of the greatest health challenges of the 21^st^ century and leads to decreased life expectancy. Among other things, it is associated with cardio-respiratory, orthopaedic, endocrine and oncologic disorders in humans and dogs^1,2^.

More than 740 million people live across 50 European countries; countries that are highly heterogeneous in terms of size, geography, climate, economy, culture and diet. The prevalence of people who are overweight or obese has reached more than 60% in many of these European countries^3^. To prevent the health-based, economic and social consequences of obesity in humans, a growing number of countries are adopting polices to prevent it from developing further^3^. Despite this, the prevalence of obesity continues to increase in both humans and pet dogs^3,4^.

Over the last decade, there has been a growing interest in using the One Health (OH) approach in health systems and research, by enforcing transdisciplinary strategies to achieve better stakeholder engagement, sustainable disease prevention and mitigation solutions^5^. The benefits of OH include improvements in animal and human health and well-being and a higher quality or larger quantity of relevant information leading to more economically efficient reasearch^5^.

Dog owners represent a suitable focus population for evaluating obesity from an OH perspective^6^, because they enables us to investigate interactions and common factors associated with and driving obesity in dogs and their owners. To date, dog-ownership has mainly been investigated as a tool to improve the status of human health^7^. An example is a weight-loss programme aimed at children and involving pet dogs, driven by the non-governmental organisation Caovida (www.caovida.com) in Portugal - a country with a severely increasing prevalence of obesity in humans^8^. Based on previous studies^9,10^, Sandøe *et al*.^7^ also argued that alongside socioeconomic, genetic and physical explanations, the nature of the human-animal bond is an important factor in the suggested correlation between obesity in people and their dogs. This is governed by psychological mechanisms related to awareness and denial that may well constitute an important underlying driver of obesity development. Obesity needs to be addressed at societal level, rather than being seen as an individual problem; in other words, obesity in humans and dogs is a real OH challenge. Large-scale studies based on OH research principles are therefore needed to better understand the links between human and canine obesity, which may well differ among countries with different demographics and cultures. The owners’ perception of obesity in themselves and their dogs also requires further investigation in the search for potential ways to improve the prevention and control of obesity.

The objectives of this study were therefore to: (1) investigate dog owner perceptions about obesity among themselves and their dogs, and (2) identify common factors and possible social, environmental and economic drivers that could relate to obesity in dog owners and their dogs.

## Results

A total of 3,418 responses were received. Of these, 3,185 from 10 European countries were eligible for inclusion, since Turkey was excluded because too few responses were received (n = 47). In addition, 186 surveys were excluded because: (a) the respondent’s reported age was below 18 years, or age information was missing (n = 64); (b) duplicated responses due to informatics errors (n = 121); or (c) an incomplete response (n = 1).

A large proportion of participants became aware of the study through social media (42%). Other responses were obtained via sharing among friends, family members or colleagues (23%), through the researchers involved in the study (19%), collaborations with or through veterinary clinics (12%) or other (4%) means (Supplementary Info 2).

### Sample characteristics

The participants were all dog owners aged between 18 and 98 years, 19.2% were men and 80.8% were women. The dogs were aged between 0.1 and 22 years, 49.5% were male and 50.5% were female. Detailed descriptive data of the participants and their dogs are available in Table 1 and Supplementary Info 2.

**Table 1:** Detailed descriptive data of the participants and their dogs.

Owner Data	Total number	%
**Man/Woman**	612/2573	19.2/80.8
**Age range in years**	18–98	
**BMI**, **mean (SD)**	24.4 (5.1)	
**Number of family members living with pet**	3 (1–10)	
**Educational level of person responding to questionnaire**		
o Primary	96	3.0
o Secondary/High School	669	21.0
o Vocational training	413	13.0
o University degree	1,583	49.7
o Postgraduate qualifications	356	11.2
o Other:	69	2.1
Employment*		
o Student	712	22.4
o Employed	2,029	63.7
o Retired	196	6.2
o Unemployed	196	6.2
o Other:	46	1.4
**Monthly family income**		
o <=national minimum wage (MW)	454	14.3
o 1–2 MW	741	23.3
o 2–3 MW	761	23.9
o 3–4 MW	681	21.4
o =>4 MW	548	17.2
**During the past 30 days (1 month) on how many days did you smoke?**		
o Every day or almost every day	652	20.5
o Some days	159	5.0
o Non- smoker	2,374	74.5
**Disease**, **Yes/No**	879/2,306	27.6/72.4
**Treatment**, **Yes/No**	674/2,511	21.2/78.8
**Dog Data**	**Total number**	**%**
**Sex**, **Female/Male**	1,577/1,608	49.5/50.5
**Age range in years**	0.1–22.0	
**BCS**, **mean (SD)**	3.1 (0.6)	
**BFI**, **mean (SD)**	27.7 (9.5)	
**Breed (5 most repeated)**	Mixed breed (n = 414), Labrador Retriever (n = 251), Golden Retriever (n = 107), German Shepherd (n = 92), Border Collie (n = 62)	Mixed breed (13.0%), Labrador Retriever (7.9%), Golden Retriever (3.4%), German Shepherd (2.9%), Border Collie (1.9%)
**Reproductive status:**		
o Intact	1866	58.6
o Neutered at < 6 months	130	4.1
o Neutered at 6–11 months	266	8.4
o Neutered at 1 year	95	3.0
o Neutered at 1–2 years	254	8.0
o Neutered at 2–8 years	441	13.8
o Neutered at > 8 years	109	3.4
Not sure	24	0.8
**Disease**, **Yes/No**	617/2,568	19.4/80.6
**Treatment**, **Yes/No**	510/2,675	16.0/84.0
**Visits to vet during last year**, **because of health problems**	0–50	

### Weight group distributions

Body mass Index (BMI) calculations (from self-reported weight and height data) indicated that 6% of respondents were underweight, 62% were of a normal weight and 32% were overweight/obese. According to the body condition scores (BCS) assessed by the owners, 22% of the dogs were overweight/obese, while according to the body fat index (BFI) chart this proportion was 56%. The agreement between the BCS and BFI categories is presented in Supplementary Info 3. When countries were grouped according to Gross Domestic Product (GDP), the highest rates of overweight/obese dog owners were found in countries with either Low or Very High GDP. In contrast, the rate of overweight/obese dogs was higher overall in countries with Low, Medium and High GDP and the lowest rates were reported in countries with Very High GDP (Table 2).

**Table 2:** Percent of underweight, normal weight and overweight/obese humans and dogs in different countries and in total.

GDP Group	Country	Humans, %				Dogs, %			
		Underweight (BMI <= 18.5)	Normal weight (BMI 18.5–25)	Overweight/obese (BMI => 25)	Underweight (BCS 1–2)	Normal weight (BCS 3)	Overweight/obese (BCS 4–5)	Normal weight (BFI 20%)	Overweight/obese (BFI 30–70%)
Low	Croatia	3.0	59.9	37.0	9.1	70.4	20.6	45.7	54.3
	Romania	10.6	58.7	30.7	7.8	64.7	27.5	33.5	66.5
	Serbia	3.1	66.7	30.2	8.8	60.0	31.3	40.5	59.5
Medium	Lithuania	7.4	64.0	28.6	15.3	63.8	20.8	30.6	69.4
	Poland	6.4	70.8	22.8	18.9	55.7	25.4	42.4	57.6
	Portugal	5.4	72.8	21.8	9.8	60.1	30.2	41.9	58.1
High	Spain	4.8	51.5	33.7	12.0	63.9	24.1	37.5	62.5
	Italy	10.3	70.6	19.1	20.6	57.9	21.4	40.5	59.5
Very High	Denmark	2.6	48.6	48.8	14.6	71.0	14.4	58.3	41.7
	Sweden	2.9	53.9	43.2	18.1	75.9	6.0	68.2	31.8
Total		**5**.**7**	**61**.**8**	**31**.**6**	**13**.**5**	**64**.**3**	**22**.**2**	**43**.**9**	**56**.**1**

Chi-square analysis showed statistically significant differences (P < 0.05) in the distribution of underweight, normal weight and overweight/obese individuals (humans and pets) across different GDP (gross domestic product) groups.

### Factors associated with being overweight/obese

Table 3 shows the variables associated with an increased probability of dog owners being overweight: aging, being a woman, and having a disease. In contrast, increasing positive attitudes towards sports and a healthy diet decreased the likelihood of being overweight/obese.

**Table 3:** Final multivariable logistic regression (Forward-Wald) model of factors associated with obesity in humans.

Risk factor	β (SE)	OR (95% CI OR)^a^	*P*-value
**Age** [per year increase]	0.04 (0.005)	1.24 (1.08–1.35)	<0.001
**Gender** (women vs. men)	0.885 (0.117)	2.42 (1.93–3.05)	<0.001
**Attitude to physical activity** [per 1 increase on Likert scale]	−1.513 (0.315)	0.22 (0.12–0.41)	<0.001
**Do you suffer from any disease?** (Yes vs. No)	0.494 (0.13)	1.64 (1.27–2.11)	<0.001
**Attitude towards diet** [per 1 increase on Likert scale]	−1.662 (0.441)	0.19 (0.08–0.451)	<0.001

Variable levels in brackets after the categorical variables describe which groups were compared in the analysis. For non-categorical variables, the estimates (β) and OR-values correspond to one unit increase in the variable. The units are given in hard brackets after the variable name. Model chi-square = 228.17. df = 6. P =< 0.001. Nagelkerke R^2^: 0.28.

^a^The reference level used for the outcome of the multivariable logistic regression is human with a normal weight.

Table 4 shows variables that discriminated significantly between *underweight* (BCS 1-2) and *normal weight* (BCS 3) dogs, according to the owner-estimated BCS. This included the age of the dog (being *underweight* was more likely in younger dogs), the type of housing (being *underweight* was more likely in dogs that lived in rural zones and spent almost all day outside, compared to dogs mostly living in a house with access to a garden), the type of diet (dogs fed home-made food were more prone to being *underweight* compared to dogs receiving a mixed diet). Finally, being *underweight* was markedly more common in dogs that were intact compared with those that were neutered.

**Table 4:** Final multinomial logistic regression (forward-Wald) models of the underweight and overweight/obesity-associated factors in dogs according to owners-estimated body condition score (BCS).

	β (ET)	OR (95%CI OR)^a^	*P*-value
**Underweight (BCS 1–2)**			
**Dog age** [per year increase]	−0.051 (0.024)	0.95 (0.91–0.99)	0.033
**Reproductive status**			
Neutered at ≥12 months	Ref.		
Intact	0.412 (0.186)	1.51 (1.05–2.18)	0.027
Neutered at <11 months	0.136 (0.266)	1.15 (0.68–1.93)	0.61
**Household**			
House/apartment with access to garden	Ref.		
Rural zone; almost the whole day outside	0.419 (0.207)	1.52 (1.01–2.28)	0.043
Rural zone; almost the whole day in the house	0.247 (0.16)	1.28 (0.94–1.75)	0.122
**Diet**			
Mixed	Ref.		
Home-made food/ Food scraps	0.522 (0.257)	1.67 (1.02–2.79)	0.042
Commercial pet food	0.118 (0.171)	1.13 (0.81–1.57)	0.491
**Overweight/obese (BCS 4–5)**			
**GDP** [per gross domestic product increase]	−0.01 (0.003)	0.99 (0.97–0.99)	0.002
**Number of family members**	0.164 (0.057)	1.18 (1.05–1.32)	0.004
**Monthly family income** [per increase in national minimum wage]	−0.141 (0.056)	0.87 (0.78–0.97)	0.011
**Influence of taking care of the pet on owner’s physical activity** (No vs. Yes)	0.406 (0.158)	1.50 (1.10–2.05)	0.01
**Dog age** [per year increase]	0.051 (0.02)	1.05 (1.01–1.10)	0.011
**Number of meals per day** [per number increase]	−0.221 (0.104)	0.80 (0.654–0.98)	0.034
**Daily exercise** [per hour increase]	−0.265 (0.078)	0.77 (0.66–0.90)	0.001
**Time spent with a pet each day** [per hour increase]	−0.143 (0.055)	0.87 (0.78–0.97)	0.009
**Sharing food with the dog while eating** (Yes vs. No)	0.253 (0.063)	1.29 (1.14–1.46)	<0.001
**Reproductive status**			
Neutered at ≥ 12 months	Ref.		
Intact	−0.995 (0.152)	0.37 (0.28–0.50)	<0.001
Neutered at < 11 months	−0.485 (0.207)	0.62 (0.41–0.92)	0.019
**Pet food rewards** (None vs. Some)	0.469 (0.166)	1.60 (1.15–2.21)	0.005
**Physical activity** (None vs. Some)	0.705 (0.309)	2.02 (1.10–3.71)	0.023

Variable levels in brackets after the categorical variables describe which groups were compared in the analysis. For non-categorical variables, the estimates (β) and OR-values correspond to one unit increase in the variable. The units are given in hard brackets after the variable name. Model chi-square = 404.33. df = 96. *P* =< 0.001. Nagelkerke *R*^2^: 0.22.

^a^The reference level used for the outcome of the multivariable logistic regression is dog with normal weight.

On the other hand, the variables that discriminated significantly between *normal weight* (BCS 3) and *overweight* (BCS 4-5) dogs, and were associated with dogs being overweight/obese were increasing age of the dog, being neutered, low family income, low GDP of the country, high number of family members, reduced number of meals per day and a short duration of exercise per day. Dogs were more prone to be overweight/obese if their owner spent little time with them, if they shared their owner’s food, if they did not receive treats or if the owner considered that having a dog did not influence his/her physical activity.

The variables that related to an increased BFI in dogs are shown in Table 5. In addition to the factors identified in relation to the reported BCS (Table 4), the type of diet, gender (males were more susceptible than females) and the owner´s unhealthy habits (e.g. smoking) were associated with an increased risk of canine obesity. Furthermore, dogs with owners who believed that obesity was not a disease, stated that their pets became ill easily and/or claimed that their dog was not happy were more likely to be overweight/obese.

**Table 5:** Final model of the logistic regression (forward-Wald) analysis determining risk factors for dog-obesity according to owners estimated BFI.

Risk factor	β (SE)	OR (95%CI OR)^a^	*P*-value
**GDP** [per gross domestic product increase]	−0.013 (0.002)	0.99 (0.98–0.99)	<0.001
**Age of the owner** [per year increase]	0.008 (0.004)	1.16 (1.08–1.21)	0.046
**Gender** (Women vs. Men)	−0.218 (0.107)	0.80 (0.65–0.99)	0.042
**Number of family members**	0.09 (0.042)	1.09 (1.01–1.19)	0.032
**Owner’s attitude to sports** [per 1 increase on Likert scale]	−0.582 (0.26)	0.56 (0.34–0.93)	0.025
**Smoking** (Yes vs. No)	0.275 (0.106)	1.32 (1.1–1.62)	0.009
**Influence of taking care of the pet on owner’s physical activity** (No vs. Yes)	0.248 (0.123)	0.78 (0.61–0.99)	0.044
**Consideration that obesity is a disease in humans** (Yes vs. No)	−0.292 (0.127)	0.75 (0.58–0.96)	0.021
**Dog age** [per year increase]	0.089 (0.015)	1.10 (1.06–1.13)	<0.001
**Sex of the dog** (Female vs. Male)	−0.29 (0.097)	0.75 (0.62–0.91)	0.003
**Pet becomes ill easily** [per 1 increase on Likert scale]	0.219 (0.055)	1.25 (1.12–1.39)	<0.001
**Belief that the pet is happy** [per 1 increase on Likert scale]	−0.236 (0.079)	0.79 (0.68–0.92)	0.003
**Reproductive status**			
Intact	Ref.		
Neutered at <11 months	0.528 (0.149)	1.70 (1.27–2.27)	<0.001
Neutered at ≥12 months	0.685 (0.117)	1.98 (1.58–2.50)	<0.001
**Diet**			
Mixed	Ref.		
Home-made food/Food scraps	−0.678 (0.202)	0.51 (0.34–0.75)	0.001
Commercial pet food	−0.23 (0.112)	0.80 (0.64–0.99)	0.039
**Daily exercise [**per hour increase**]**	−0.141 (0.062)	0.87 (0.77–0.98)	0.022

Variable levels in brackets after the categorical variables describe which groups were compared in the analysis. For non-categorical variables the estimates (β) and OR-values correspond to one unit increase in the variable. The units are given in hard brackets after the variable name. Model chi-square = 357.41. df = 17. *P* =< 0.001. Nagelkerke *R*^2^: 0.21.

^a^The reference level used for the outcome of the multivariable logistic regression is dog with normal weight.

### Perception of obesity by dog owners

The proportion of respondents who did not consider obesity to be a disease differed across countries, varying between 3% and 46% for those who did not believe obesity to be a disease in humans and between 2% and 49% for those who did not believe obesity to be a disease in dogs (Table 6).

**Table 6:** Percent of respondents who agreed or disagreed that obesity is a disease in humans and/or dogs, and who believed interdisciplinary collaboration either would or would not be useful in combatting obesity.

	Human obesity is a disease		Canine obesity is a disease		Collaboration	
	Agreed	Not agreed	Agreed	Not agreed	Yes	No
Croatia	92	8	89	10	68	25
Denmark	46	43	43	49	38	28
Italy	95	5	94	5	89	7
Lithuania	76	20	83	13	64	17
Poland	96	6	93	7	93	7
Portugal	97	3	98	2	86	4
Romania	97	3	98	2	71	29
Serbia	88	10	84	13	48	22
Spain	97	3	96	4	82	15
Sweden	58	34	56	40	45	39

Owners most often mentioned lifestyle (a sedentary way of life) and food-related factors (eating fast food, high content of fats and sugar, additives, processed food) as reasons for the development of obesity. These were, followed by psychological factors (stress, depression, demotivation, laziness, poor self-discipline, and over-humanisation of pet dogs) and the modern way of life (every day rush, limited time to care for oneself and one’s pets).

The main recommendations proposed by owners to stop the increase of obesity in both humans and dogs were summarised into three topics:

#### Socio-economic

increased social education/awareness/knowledge about the risks of obesity and associations between diet, exercise and health for humans and dogs through easily accessible courses, TV and radio programmes. Better health care assistance for weight loss and maintenance for humans and dogs and better psychiatric monitoring, as well as implementation of food legislation and more government control over the food industry (e. g. taxes on unhealthy food/sugar).

#### Lifestyle

reinforce/implement physical exercise within urban areas (at work or school, during leisure time, in daily life, making room for dogs in city life).

#### Food

Easily accessible healthy and balanced meals for humans and dogs.

Finally, in most countries, a high proportion (up to 93%) of owners reported that they believed cooperation between human and veterinary health care professionals (including scientists) would be an important factor in ensuring comprehensive education about the importance of healthy eating and physical activity for people and their pets, and that this in turn would help prevent obesity (Table 6). However, on average, 19% of participants could not see any connection between the disciplines, or believed it would be difficult to carry out such collaboration. Despite this, many of these participants stated that specialists from both disciplines should concentrate more on obesity counselling.

## Discussion

This study is the first to investigate factors relating to self-reported obesity rates in both dog owners and their dogs across ten different European countries. The human obesity rates reported in the present study were lower than those reported by WHO^11^, with the exception of data obtained from Denmark and Sweden, which showed rates similar to those reported for women^11^. In fact, the majority of respondents in these two countries were women (91% and 95%, respectively). One possible reason for the lower obesity rates in our study could be inaccuracy in self-reported data, yet WHO also used on self-reported data in the majority of the countries^11^. Another reason could be that the study population consisted only of dog owners, who may be leaner than the general population. It is important to be aware that the survey was answered voluntarily. The sample could therefore be biased relative to the general dog owner population in the participating countries and the comparison to WHO data should be interpreted with caution. Although the scientific basis is weak and further investigation is required, dog ownership has been related to increased physical activity, decreased obesity rates, general health benefits and lower mortality than the general population^12–17^. Owning a dog has been suggested as a means to combat a sedentary lifestyle through enhanced motivation for activity^18^. Furthermore, companion dogs have been shown to support both social and physical activity during a weight-loss period in humans^19^. It could therefore be speculated that dog ownership could contribute to the many complex interactions affecting the risk of obesity in humans^15^. However, the mean percentage of dog owners who were overweight/obese was quite high (19.1–48.8%) across the different countries (although still lower than reports from WHO), indicating that owning a dog does not necessarily protect people from becoming overweight.

The economic transition towards greater GDP is linked to demographic (aging population, urbanisation), epidemiological or health (increase in non-communicable diseases), technological (mechanisation) and nutritional (more processed, high-energy foods) changes that together might result in an obesogenic environment^20^. Obesity could be described as the result of people responding normally to an obesogenic environment. This could explain why the highest rates of human obesity were observed in countries with the highest GDP, and would support government interventions and investment in monitoring and research programmes in order to reduce the risk of obesity^21^. However, it is important to take into account that other confounders could play a role. For instance, the two countries with the highest GDP were both North European countries, and latitude was not considered in this study. It is therefore possible that climate-related bias could exist.

Different obesity rates were reported in dogs, depending on which morphological scale was used (BCS or BFI). Variation in reported obesity rates could be due to differences in the specific scales and descriptions of the different scores. The BCS scale covers the whole range of body compositions from underweight (body fat percentage (BF%) < 10) to obese (BF% = 45), while the BFI covers normal weight (BF% = 20) to morbidly obese (BF% = 70). In the BCS system, overweight (BCS 4/5) is described as heavy or stout, while overweight in the BFI system BFI30 is described as having a waist and tail base combined with moderate fat cover^22^. The different interpretations were also observed in our data where 939 dogs classified as normal weight by the BCS system were considered overweight in the BFI system (Supplementary Info 3). It is very likely that some of these dogs were in fact slightly overweight. Furthermore, although the two scales were previously validated using standard physical methods (the deuterium oxide [D2O] dilution method in case of BCS^23^, and dual-energy x-ray absorptiometry [DEXA] in case of BFI^22^), Witzel *et al*.^24^ suggest that because the BFI has more categories for overweight/obese dogs, it would be more accurate than the 5-point BCS method for estimating the body fat percentage in overweight and obese dogs particularly those with a BF% > 45. However, both scales are widely used in canine obesity research and complement each other in terms of the BF% range covered. Therefore, we find it is important to highlight how the use of different scales can influence research results and recommend that investigators choose the best suitable method based on their research questions.

Despite the differences between the scales and risk of inaccurate estimation by owners, the recorded occurrence of overweight/obese dogs is in accordance with previously reported canine obesity rates (16–62%) in European countries^10,25–29^. However, the results cannot be directly compared due to different study populations (e.g. shelter dogs in Italy^26^ and an obesogenic area in Spain^29^) and different methods used to evaluate canine obesity occurrence. Based on the assumption that possible owner bias is constant and independent of nationality, it can be postulated that this is the first study to compare the occurrence of obesity in the general canine population across a number of European countries.

In the present study, there was no direct correlation between the prevalence of obesity in owners and dogs. These findings are in contrast to previously reported studies, which showed that overweight/obese people were more likely to own overweight/obese dogs^9,30^. This discrepancy could be explained by the different populations studied (single vs. multiple countries) and the methods used (*in situ* vs. anonymous questionnaire). Furthermore, in the present study, the highest overweight/obesity rates among the dog owners were observed in countries with the highest GDP and, interestingly, the participants from these countries reported their dogs to have the lowest BCS and BFI. Various hypotheses could explain this apparent paradox. Firstly, in countries with high GDP, people may be more attentive to the health and well-being of their pets, and may be economically able to treat and care for their animals adequately. This can be reinforced by the comments of some Swedish survey participants, such as - “*I´m aware that I´m overweight and what consequences this may have*. *This should not happen to my dog*!”. In addition, the Swedish participants were mostly recruited through a Facebook group with special interests in animal heath, which might partly explain the low reported number of overweight dogs. However, it has previously been postulated that dog owners with higher incomes could be more prone to underestimate the body condition of their pet^31^.

In this study, the main drivers of obesity among dog owners and their pets were of a social nature, including lack of exercise and diet-related factors. This is in accordance with previously reported findings for humans and dogs, respectively^4,32^. Furthermore, dogs owned by smokers were more prone to being overweight in this study. These observations all indicate that human values and habits influence the risk of developing obesity for both humans and their dogs. Humans are generally responsible for their own selected lifestyle, which in turn may be influenced by societal factors, such as the obesogenic environment. The dogs, however, are owner-dependent, being fully influenced by the preferences and habits of their owners^33^.

Increasing body fat mass affects the physiology and metabolism of both humans and dogs^2,4^. In accordance, the results of this study indicate that being overweight/obese was associated with an increased risk of morbidity in dog owners. In contrast, there was no direct association between dogs being overweight/obese and suffering from a disease. However, an increasing BFI was associated with the owners’ perception that their pets became ill easily, and was inversely associated with the owners believing that the dog was happy. These results are in accordance with the previously reported observations that the health-related quality-of-life (HRQoL) decreased in obese dogs^33^. It has been shown that obesity mainly diminishes the physical HRQoL component in humans^34^. An improvement in HRQoL in both humans and dogs was reported after successful weight loss^34,35^.

The potential obesity drivers and solutions to combat obesity mentioned by the majority of participants agree with the factors most often described in the scientific literature and reported by different health organisations. This indicates that dog owners generally know the basic information related to drivers of obesity and possible ways to combat it. Education regarding healthy nutrition or greater awareness of the health risks has not been associated with an improvement in human or canine obesity rates^10,36,37^. Our data indicate that owners who do not consider obesity to be a disease (due to lack of knowledge or because they consider obesity to be a nutritional or metabolic disorder) are more likely to have an overweight/obese dog. Based on this, it can be hypothesised that one way to prevent and/or even stop obesity from spreading could be better educational strategies for increased awareness and knowledge about this disease and the ways to combat it.

It has been proposed that implementing an OH approach could provide a number of benefits in comparison to ‘silo approaches’ because sharing of knowledge and experience could strengthen the message and reach a larger audience^5^. The vast majority of dog-owners agreed that collaboration between human and veterinary medicine specialists would be important in order to increase human and canine obesity-related knowledge and awareness among members of the family, and by thus prevent obesity development. However, there were a number of respondents that still did not see any benefit in transdisciplinary collaboration for obesity treatment or prevention in humans and dogs.

The main limitation of this study is related to the methodology used for the data collection. It is known that data obtained using online questionnaires might present a bias in sample selection, relatively low quality data and low response rates, and therefore may not be as accurate as a direct clinical observation and evaluation of clinical histories. Regardless of these potential drawbacks, anonymous questionnaires have been linked to more truthful responses^38^. Furthermore, the use of questionnaires is widely accepted for studies of an exploratory nature like this one^39,40^ and by international organisations aiming to create a general overview of the population such as WHO^11^. However, the recruitment procedures (Supplementary Info 2) varied widely among countries, which could have resulted in data bias.

In conclusion, the results indicate that the self-reported occurrence of obesity among dog owners may be lower than in the general human population. The main factors associated with obesity in both owners and their dogs were increasing age, poor diet and low physical activity. Furthermore, the owners that did not consider obesity to be a disease were more likely to have obese dogs. Finally, although dog owners seemed to be aware of the main drivers of obesity, a high percentage did not consider obesity to be a disease, and specific information about this subject should therefore be provided in order to combat human and canine obesity. Although the present study identified several factors related to dog-owner perceptions, actions and owner dog relationships that were specifically associated with canine obesity, further studies that can objectively assess the body condition of both owners and their dogs, as well as the validity of the respondents’ answers, are required to verify the results and identify and explain the nature of the causality of obesity in dogs and dog owners.

## Methods

### Data collection

A cross-sectional questionnaire-based study targeting dog owners across multiple countries was performed. The inclusion criteria included being an over 18 years of age and living with and taking care of at least one dog. The study was approved by the Ethical Committee of the University of Murcia (1374/2016), and by the local Ethical Committees in the participating countries, where required. All methods were performed in accordance with the relevant guidelines and regulations of each country.

The questionnaire was inspired by previously described surveys from the disciplines of human and veterinary medicine^41–46^. After the initial drafting, a pilot study was performed in Spain. In total, 57 individuals were asked to answer the questionnaire and give their direct feedback to the authors, thus enabling validation of the responses. The questionnaire was adjusted accordingly and a second pilot study was performed in Poland, where 321 responses were obtained within 2 weeks. After analysing these data, two additional questions were included to collect information about how participants became aware of the study and in which part of the country they lived, and two questions were modified – one related to smoking habits and the other to family income. In addition, it became mandatory to respond to all questions. This led to the final version of the questionnaire consisting of informed consent followed by five main sections relating to information about [1] the owners (number of questions (nq) = 32) including questions about their age, height, body weight, gender, employment status, attitude to physical activity and diet; [2] the dogs (nq = 23) including questions about breed, age, weight, sex, body condition, diet and physical activity; [3] the owner-dog relationship (nq = 12) including questions about time spent with the animal, attitudes towards feeding behaviour and the dog to sleeping in the owner’s bed; [4] the obesity background, reflecting the respondent’s perception of obesity as a societal challenge (nq = 5); [5] the respondent’s perception of the questionnaire itself (nq = 3; Supplementary Info 1). The identity of the respondents was kept anonymous.

The aim was to include at least 100 questionnaire responses from at least two representative countries from each of the different regions in Europe. This included Southern Europe: Spain, Portugal and Italy; Northern Europe: Denmark and Sweden; Eastern Europe: Lithuania, Romania and Serbia; Central Europe: Croatia and Poland. The translated questionnaires were set up online using Google Forms and links were distributed through email and social media. In addition, hard copies were distributed via veterinary clinics, personal contacts, etc. An unlimited number of responses could be collected from December 2016 to March 2017.

### Data editing for analysis

Dog owners were categorised into three groups according to their body mass index (BMI), as calculated from their self-reported weight and height using the formula:

$${\rm{BMI}}={\rm{weight}}({\rm{kg}})/{({\rm{height}}({\rm{m}}))}^{2}$$

where underweight included BMI < 18.5, normal weight was 18.5 ≤ BMI > 25, and overweight/obese was BMI ≥ 25.

The body condition of the dogs was assessed by the owners, based on the provided body condition score (BCS) and body fat index (BFI) charts (see Supplementary Material 1, questions 38 and 39). Dogs were grouped according to the BCS as underweight (BCS, 1 and 2), normal weight (BCS, 3), or overweight/obese (BCS, 4 and 5); and according to the BFI as normal weight (BFI, 20%) or overweight to obese (BFI, 30–70%).

The countries were categorised into four groups according to the Gross Domestic Product (GDP) based on the 2016 Eurostat data (http://ec.europa.eu/eurostat/tgm/table.do?tab=table&plugin=1&language=en&pcode=tec00114, accessed 9^th^ December 2017): *Group 1 (Low) –* countries with GDP < 60, which included Serbia (GDP = 36), Romania (GDP = 55) and Croatia (GDP = 59); *Group 2 (Medium) –* countries with GDP between 61 and 80, which included Poland (GDP = 68), Lithuania (GDP = 75) and Portugal (GDP = 78); *Group 3 (High) –* countries with GDP between 81 and 110, which included Spain (GDP = 91) and Italy (GDP = 96); *Group 4 (Very high) –* countries with GDP > 110, which included Sweden (GDP = 123) and Denmark (GDP = 125).

In order to summarise the dog owners’ responses related to their perception of obesity as a societal challenge, the responsible persons from each country were asked to review the written comments and report the three most often-mentioned reasons for increasing obesity rates, three recommendations to stop this increase, and the main ideas about the potential of human and veterinary healthcare professionals collaborating to combat obesity.

### Statistical analysis

Descriptive statistical analysis included calculation of the mean and standard deviation (SD) for continuous variables along with the proportion of observations in each category for categorical variables. Three multivariable logistic regression models were used to determine risk factors associated with the BMI of owners, and the BCS and the BFI of pets. A forward stepwise variable selection procedure was used to select the final model for which significant variables below a *P*-value of 0.05 were retained. The -2 log-likelihood ratio test was used to test the overall significance of the predictive equation. The significance of the variables in the model was assessed by the Wald χ^2^ test and confidence intervals. The fit of the model was assessed using the Hosmer-Lemeshow goodness-of-fit χ^2^ test. The statistical procedures were performed using standard software (Microsoft Excel, Graphpad Prism, and SPSS version 23.0 for Windows). Associations and differences among groups were considered statistically significant when *P* < 0.05.

## Electronic supplementary material

Supplementary Information

## Data Availability

The data is available upon request.

## References

[1] Weeth LP (2016). Other Risks/Possible Benefits of Obesity. Vet. Clin. North Am. Small Anim. Pract..

[2] Fruh SM (2017). Obesity: Risk factors, complications, and strategies for sustainable long-term weight management. J. Am. Assoc. Nurse Pract..

[3] WHO | Obesity and overweight. *WHO* (2018).

[4] German AJ (2006). The growing problem of obesity in dogs and cats. J. Nutr..

[5] Häsler B, Cornelsen L, Bennani H, Rushton J (2014). A review of the metrics for One Health benefits. Rev. Sci. Tech..

[6] Chandler M (2017). Obesity and Associated Comorbidities in People and Companion Animals: A One Health Perspective. J. Comp. Pathol..

[7] Sandøe P, Palmer C, Corr S, Astrup A, Bjørnvad CR (2014). Canine and feline obesity: a One Health perspective. Vet. Rec..

[8] Carmo Ido (2006). Prevalence of obesity in Portugal. Obes. Rev..

[9] Kienzle E, Bergler R, Mandernach A (1998). A comparison of the feeding behavior and the human-animal relationship in owners of normal and obese dogs. J. Nutr..

[10] Courcier EA, Thomson RM, Mellor DJ, Yam PS (2010). An epidemiological study of environmental factors associated with canine obesity. J. Small Anim. Pract..

[11] WHO | Overweight and obesity. *WHO* (2017).

[12] Jennings LB (1997). Potential Benefits of Pet Ownership in Health Promotion. J. Holist. Nurs..

[13] Cutt H, Giles-Corti B, Knuiman M, Burke V (2007). Dog ownership, health and physical activity: A critical review of the literature. Health Place.

[14] Coleman KJ (2008). Physical activity, weight status, and neighborhood characteristics of dog walkers. Prev. Med. (Baltim)..

[15] Christian HE (2013). Dog Ownership and Physical Activity: A Review of the Evidence. J. Phys. Act. Heal..

[16] Westgarth C (2017). The association between dog ownership or dog walking and fitness or weight status in childhood. Pediatr. Obes..

[17] Mubanga M (2017). Dog ownership and the risk of cardiovascular disease and death – a nationwide cohort study. Sci. Rep..

[18] Wohlfarth R, Mutschler B, Beetz A, Kreuser F, Korsten-Reck U (2013). Dogs motivate obese children for physical activity: key elements of a motivational theory of animal-assisted interventions. Front. Psychol..

[19] Kushner RF, Blatner DJ, Jewell DE, Rudloff K (2006). The PPET Study: People and Pets Exercising Together*. Obesity.

[20] Popkin BM (1998). The nutrition transition and its health implications in lower-income countries. Public Health Nutr..

[21] Swinburn BA (2011). The global obesity pandemic: shaped by global drivers and local environments. Lancet.

[22] Paetau‐Robinson I, Stiers CA, Stone BA (2017). The Body Fat Index Chart Is Equivalent to DEXA for Determination of Percent Body Fat During Weight Loss and Weight Maintenance in Dogs. J. Vet. Intern. Med..

[23] Sagawa MM, Nakadomo F, Honjoh T, Ishioka K, Saito M (2002). Correlation between plasma leptin concentration and body fat content in dogs. Am. J. Vet. Res..

[24] Witzel AL (2014). Use of a novel morphometric method and body fat index system for estimation of body composition in overweight and obese dogs. J. Am. Vet. Med. Assoc..

[25] Edney AT, Smith PM (1986). Study of obesity in dogs visiting veterinary practices in the United Kingdom. Vet. Rec..

[26] Ricci, R. *et al*. Body condition score (BCS) and metabolic status of shelter dogs. *Ital*. *J*. *Anim*. *Sci*. **6**, (2010).

[27] Corbee RJ (2012). Obesity in show dogs. J. Anim. Physiol. Anim. Nutr. (Berl)..

[28] Raffan E, Smith SP, O’Rahilly S, Wardle J (2015). Development, factor structure and application of the Dog Obesity Risk and Appetite (DORA) questionnaire. PeerJ.

[29] Montoya-Alonso, J. A. *et al*. Prevalence of canine obesity, obesity-related metabolic dysfunction, and relationship with owner obesity in an obesogenic region of Spain. *Front*. *Vet*. *Sci*. **4** (2017). 10.3389/fvets.2017.00059 PMC5403824 28487859

[30] Nijland ML, Stam F, Seidell JC (2010). Overweight in dogs, but not in cats, is related to overweight in their owners. Public Health Nutr..

[31] Courcier EA, Mellor DJ, Thomson RM, Yam PS (2011). A cross sectional study of the prevalence and risk factors for owner misperception of canine body shape in first opinion practice in Glasgow. Prev. Vet. Med..

[32] Bryan AD (2017). Behavioral and Psychological Phenotyping of Physical Activity and Sedentary Behavior: Implications for Weight Management. Obesity.

[33] Yam PS (2016). Impact of canine overweight and obesity on health-related quality of life. Prev. Vet. Med..

[34] Olszanecka-Glinianowicz M, Zygmuntowicz M, Owczarek A, Elibol A, Chudek J (2014). The impact of overweight and obesity on health-related quality of life and blood pressure control in hypertensive patients. J. Hypertens..

[35] German AJ (2012). Quality of life is reduced in obese dogs but improves after successful weight loss. Vet. J..

[36] Yaissle JE, Holloway C, Buffington CAT (2004). Evaluation of owner education as a component of obesity treatment programs for dogs. J. Am. Vet. Med. Assoc..

[37] Gordon-Larsen P (2001). Obesity-Related Knowledge, Attitudes, and Behaviors in Obese and Non-obese Urban Philadelphia Female Adolescents. Obes. Res..

[38] Ong AD, Weiss DJ (2000). The Impact of Anonymity on Responses to Sensitive Questions1. J. Appl. Soc. Psychol..

[39] Janssen I (2005). Comparison of overweight and obesity prevalence in school-aged youth from 34 countries and their relationships with physical activity and dietary patterns. Obes. Rev..

[40] Taylor MJ (2015). Measurement of the association between perceived exercise capability and childhood obesity: a feasibility study. Lancet.

[41] Robertson ID (2003). The association of exercise, diet and other factors with owner-perceived obesity in privately owned dogs from metropolitan Perth, WA. Prev. Vet. Med..

[42] Carciofi AC (2005). A weight loss protocol and owners participation in the treatment of canine obesity. Ciência Rural.

[43] McGreevy PD (2005). Prevalence of obesity in dogs examined by Australian veterinary practices and the risk factors involved. Vet. Rec..

[44] Bland IM, Guthrie-Jones A, Taylor RD, Hill J (2009). Dog obesity: Owner attitudes and behaviour. Prev. Vet. Med..

[45] Lima-Serrano M, Lima-Rodríguez JS, Sáez-Bueno Á (2012). Design and validation of scales to measure adolescent attitude toward eating and toward physical activity. Rev. Esp. Salud Publica.

[46] Mao J, Xia Z, Chen J, Yu J (2013). Prevalence and risk factors for canine obesity surveyed in veterinary practices in Beijing, China. Prev. Vet. Med..

