# Supplementary material for: European dog owner perceptions of obesity and factors associated with human and canine obesity
Source: Sci Rep. 2018 Sep 6;8:13353. doi: 10.1038/s41598-018-31532-0 (PMC6127309; doi:10.1038/s41598-018-31532-0)
Supplement: Supplementary file 1 — Supplementary Information [file 41598_2018_31532_MOESM1_ESM.pdf]

European dog-owner perceptions of obesity and factors associated with human and canine obesity

Alberto Muñoz-Prieto,[1] Liza Rosenbaum Nielsen,[2] Roman Dąbrowski,[3] Charlotte Reinhard Bjørnvad,[4] Josefin Söder,[5] Elsa Lamy,[6] Ingrida Monkeviciene,[7] Blanka Beer Ljubić,[8] Iosif Vasiu,[9] Sara Savich,[10] Francesca Busato,[11] Zeki Yilmaz,[12] Antonio F. Bravo-Cantero,[13] Malin Öhlund,[14] Sónia Lucena,[6] Rasa Zelvyte,[7] Jasna Aladrovic,[15] Pia Lopez-Jornet,[16] Marco Caldin,[11] Catarina Lavrador,[6] Birute Karveliene,[17] Vladimir Mrljak,[8] Jovita Mazeikiene,[18] Asta Tvarijonaviciute.[1]

1. Interlab-UMU, Regional Campus of International Excellence "Mare Nostrum", University of Murcia, Ed.16, 4a planta, Espinardo, Murcia 30100, Spain.
2. Department of Veterinary and Animal Sciences, Faculty of Health and Medical Sciences, University of Copenhagen, Grønnegårdsvej 8, 1870 Frederiksberg C, Denmark.
3. Department and Clinic of Animal Reproduction, Faculty of Veterinary Medicine, University of Life Sciences, Gleboka 30 St, 20-612 Lublin, Poland.
4. Department if Veterinary Clinical Sciences, Dyrlægevej16, 1870 Frederiksberg C University of Copenhagen, Denmark.
5. Department of Anatomy, Physiology and Biochemistry, Swedish University of Agricultural Sciences, P.O. Box 7011, SE_750 07 Uppsala, Sweden.
6. Instituto de Ciências Agrárias e Ambientais Mediterrânicas (ICAAM), Departamento de Medicina Veterinária, Escola de Ciências e Tecnologia, Universidade de Évora, Núcleo da Mitra, Apartado 94, 7006-554 Évora, Portugal.

7. Department of Anatomy and Physiology, Research Center of Digestive Physiology and Pathology, Veterinary Academy, Lithuanian University of Health Sciences, Tilzes 18, LT-47181 Kaunas, Lithuania.

8. Clinic for Internal Diseases, Faculty of Veterinary Medicine, Heinzelova 55, 10000 Zagreb, Croatia.

9. Small Animal Emergency Hospital, University of Agricultural Sciences and Veterinary Medicine Cluj-Napoca, Calea Mănăştur 3-5, 400372, Romania.

10. Scientific Veterinary Institute "NoviSad", Serbia.

11. San Marco Veterinary Clinic, Padova, Italy.

12. Department of Internal Medicine, Faculty of Veterinary Medicine, Uludag University, 16059, Nilufer, Bursa, Turkey.

13. Estadistica Murcia, 30565 Las Torres de Cotillas, Murcia, Spain.

14. Department of Clinical Sciences, Swedish University of Agricultural Sciences, Box 7054, 750 07 Uppsala, Sweden.

15. Department of Physiology and Radiobiology, Faculty of Veterinary Medicine, Heinzelova 55, 10000 Zagreb, Croatia.

16. Department of Oral Medicine, Faculty of Medicine, University of Murcia, 30100 Espinardo, Spain.

17. Veterinary faculty, Dr. L Kriaučeliūnas Small Animal Clinic (teaching hospital), Veterinary Academy, Lithuanian University of Health Sciences, Tilzes 18, LT-47181 Kaunas, Lithuania.

18. InMedica Vilnius - Alfa Clinic, Baltrusaicio 3, 06120 Vilnius, Lithuania.

*** Correspondence to**: Dr. Asta Tvarijonaviciute, Interlab-UMU, Regional Campus of International Excellence "Mare Nostrum", University of Murcia, Ed.16, 4a planta, Espinardo, Murcia 30100, Spain. Email: asta@um.es Tel. +34868887168

Supplementary Information 1. The final version of the questionnaire used in the study and the comments related to the statistical approach of some of the questions.

Obesity among pet-owners and pets

Purpose: To study the possible causes of obesity and associated diseases among pet owners and their pets.

- All personal data will be protected with guaranteed confidentiality in accordance with Law 15 of 13th December 1999.

**Number**	**Question**	**Comments**
**Informed consent**	**□ I understand that all data supplied by myself in this questionnaire will only be used for the purposes specified above.**	
**1**	**Where did you hear about this study?**<br>• Through a researcher involved in the study<br>• Through the veterinary clinic I go to with my pet<br>• Through somebody else who went to a veterinary clinic<br>• Through a dog activity or dog breeding organisation<br>• Through a colleague<br>• Through a friend or family member<br>• Through social media (e.g. Facebook, Twitter)<br>• Other	
**2**	**In what part of the country do you currently live?**<br>• South<br>• North<br>• Eastern<br>• Western<br>• Central<br>• Do not know	
	**Owner data**<br>Criteria: - If more than one person is taking care of the same pet, the form must be filled in by only one individual	
**3**	Age (years)	
**4**	Gender<br>o Man<br>o Woman	
**5**	Height (cm)	
**6**	Weight (kg)	
**7**	Number of family members living with pet *<br>1 | 2 | 3 | 4 | 5 | 6 | 7 | 8 | 9 | 10	
**8**	Educational level of person responding to questionnaire*<br>o Primary	Because of low number of responses for

<table>
<tr><td></td><td>Secondary/High School
Vocational training
University degree
Postgraduate qualifications
Other</td><td>Primary and Secondary education levels, these were fused for further statistical analysis.</td></tr>
<tr><td>9</td><td>Employment *
Student
Employed
Retired
Unemployed</td><td></td></tr>
<tr><td>10</td><td>Monthly Family income *
=< national minimum wage (MW)
1 – 2 MW
2 – 3 MW
3 – 4 MW
=> 4 MW</td><td></td></tr>
<tr><td>11-17</td><td>Attitude toward physical activity
Please respond to the following statements with a score of 1-5 where 1 = total disagreement and 5 = total agreement. Please tick only one box in each row; if it is difficult to choose only one answer, choose the option that reflects your opinion most of the time*

Doing sport makes me feel good *
<table><tr><td>1</td><td>2</td><td>3</td><td>4</td><td>5</td></tr><tr><td>Total disagreement</td><td></td><td></td><td></td><td>Total agreement</td></tr></table>
I like doing one or more of the following activities on a regular basis (at least once a week): walking, running, sports, etc. *
<table><tr><td>1</td><td>2</td><td>3</td><td>4</td><td>5</td></tr><tr><td>Total disagreement</td><td></td><td></td><td></td><td>Total agreement</td></tr></table>
I get bored doing sports activities
<table><tr><td>1</td><td>2</td><td>3</td><td>4</td><td>5</td></tr><tr><td>Total disagreement</td><td></td><td></td><td></td><td>Total agreement</td></tr></table>
Physical activity relieves stress
<table><tr><td>1</td><td>2</td><td>3</td><td>4</td><td>5</td></tr><tr><td>Total disagreement</td><td></td><td></td><td></td><td>Total agreement</td></tr></table>
I do not like physical activity because I feel very tired
<table><tr><td>1</td><td>2</td><td>3</td><td>4</td><td>5</td></tr><tr><td>Total disagreement</td><td></td><td></td><td></td><td>Total agreement</td></tr></table>
I do not have the self-discipline necessary to do exercise *
<table><tr><td>1</td><td>2</td><td>3</td><td>4</td><td>5</td></tr><tr><td>Total disagreement</td><td></td><td></td><td></td><td>Total agreement</td></tr></table>
I like physical activity because it is beneficial to health
<table><tr><td>1</td><td>2</td><td>3</td><td>4</td><td>5</td></tr><tr><td>Total disagreement</td><td></td><td></td><td></td><td>Total agreement</td></tr></table>
</td><td>The responses for questions 11, 12, 14, 17 were scored from 0 to 1 (1=0, 2=0.25, 3=0.5, 4=0.75, and 5=1) and the responses 13, 15, and 16 were scored from 1 to 0 (1=1, 2=0.75, 3=0.5, 4=0.25, and 5=1). Then the mean score was calculated for further statistical analysis.</td></tr>
<tr><td>18</td><td>During the past 30 days (1 month) on how many days did you</td><td>For statistical analysis,</td></tr>
</table>

<table>
<tr><td></td><td>smoke?
o Every day or almost every day
o Some days
o Not smoker</td><td>responses to “Every day or almost every day” and “some days” were fused.</td></tr>
<tr><td>19</td><td>Do you suffer from any disease *
o No
o Yes</td><td></td></tr>
<tr><td>20</td><td>Which disease(s) do you suffer from?</td><td></td></tr>
<tr><td>21</td><td>Are you receiving chronic treatment (> 1 month duration)?
o Yes
o No</td><td></td></tr>
<tr><td>22</td><td>What treatment(s) do you receive?</td><td></td></tr>
<tr><td>23-32</td><td>Attitude towards diet
Please respond to the following statements with a score of 1-5 where 1 = total disagreement and 5 = total agreement. Please tick only one box in each row; if it is difficult to choose only one answer, choose the option that reflects your opinion most of the time*

I believe eating a healthy diet is important for my overall health
<table><tr><td>1</td><td>2</td><td>3</td><td>4</td><td>5</td></tr><tr><td>Total disagreement</td><td></td><td></td><td></td><td>Total agreement</td></tr></table>
Home-made food is better than fast-food or ready-prepared food
<table><tr><td>1</td><td>2</td><td>3</td><td>4</td><td>5</td></tr><tr><td>Total disagreement</td><td></td><td></td><td></td><td>Total agreement</td></tr></table>
I feel better eating a healthy diet
<table><tr><td>1</td><td>2</td><td>3</td><td>4</td><td>5</td></tr><tr><td>Total disagreement</td><td></td><td></td><td></td><td>Total agreement</td></tr></table>
Healthy food is boring
<table><tr><td>1</td><td>2</td><td>3</td><td>4</td><td>5</td></tr><tr><td>Total disagreement</td><td></td><td></td><td></td><td>Total agreement</td></tr></table>
Healthy meals are laborious (more difficult to shop for, prepare, transport, etc.)
<table><tr><td>1</td><td>2</td><td>3</td><td>4</td><td>5</td></tr><tr><td>Total disagreement</td><td></td><td></td><td></td><td>Total agreement</td></tr></table>
It is normal to skip breakfast
<table><tr><td>1</td><td>2</td><td>3</td><td>4</td><td>5</td></tr><tr><td>Total disagreement</td><td></td><td></td><td></td><td>Total agreement</td></tr></table>
How food tastes is more important than its health benefit
<table><tr><td>1</td><td>2</td><td>3</td><td>4</td><td>5</td></tr><tr><td>Total disagreement</td><td></td><td></td><td></td><td>Total agreement</td></tr></table>
I do not have the self-discipline to follow a healthy diet
<table><tr><td>1</td><td>2</td><td>3</td><td>4</td><td>5</td></tr><tr><td>Total disagreement</td><td></td><td></td><td></td><td>Total agreement</td></tr></table>
A healthy diet includes a variety of foods (cereals, vegetables, meat, etc.)</td><td>The responses for questions 23, 24, 25, 32 were scored from 0 to 1 (1=0, 2=0.25, 3=0.5, 4=0.75, and 5=1) and for questions 26-31 were scored from 1 to 0 (1=1, 2=0.75, 3=0.5, 4=0.25, and 5=1). Then the mean score was calculated for further statistical analysis.</td></tr>
</table>

<table>
<tr><td></td><td><table><tr><td>1</td><td>2</td><td>3</td><td>4</td><td>5</td></tr><tr><td>Total disagreement</td><td></td><td></td><td></td><td>Total agreement</td></tr></table></td><td></td></tr>
<tr><td></td><td>Pet data
Criteria: - If you have more than one pet, you must apply the questionnaire to just one animal.</td><td></td></tr>
<tr><td>33</td><td>Breed (Please chose one of the list)</td><td>The owners were provided by the list of dog breeds and the option “other” was also available.</td></tr>
<tr><td>34</td><td>Age (years)</td><td></td></tr>
<tr><td>35</td><td>Sex
o Male
o Female</td><td></td></tr>
<tr><td>36</td><td>Reproductive status
o Intact
o Neutered at <6 months
o Neutered at 6-11 months
o Neutered at 1 year
o Neutered at 1-2 years
o Neutered at 2-8 years
o Neutered at >8 years
o Not sure</td><td>For statistical analysis, responses to were grouped to intact, neutered ≤11 months, and neutered >1 year were fused.</td></tr>
<tr><td>37</td><td>Weight (kg)</td><td></td></tr>
<tr><td>38</td><td>On the basis of the images below: what score corresponds to your pet?
<table><tr><td>1</td><td>2</td><td>3</td><td>4</td><td>5</td></tr><tr><td>Very thin</td><td></td><td></td><td></td><td>Obese</td></tr></table></td><td>The body condition chart, available at http://cavalierhealth.org/images/dog_body_condition_scoring_chart.jpg, was included to help owners to score his/her dogs body condition.</td></tr>
<tr><td>39</td><td>Given the image below: what body fat index corresponds to your pet?
<table><tr><td>20%</td><td>30%</td><td>40%</td><td>50%</td><td>60%</td><td>70%</td></tr></table></td><td>The Hill´s body fat index (BFI) risk chart, available at http://www.assaro.c</td></tr>
</table>

		o.za/uploads/1/0/3/8/10383298/3840090_orig.png, was included to help owners to assess his/her dogs BFI
**40**	Household<br>o Rural zone; the dog spends almost the whole day outside<br>o Rural zone; the dog spends almost the whole day in the house<br>o House/apartment without access to garden<br>o House/apartment with access to garden<br>o Other	
**41**	Diet<br>o Home-made food<br>o Food scraps<br>o Commercial pet food<br>o Mixed<br>o Other	For statistical analysis, responses “Home-made food” and “Food scarps” were fused
**42**	Number of meals per day<br>o 1<br>o 2<br>o 3<br>o > 3	
**43**	How do you calculate the amount of food for your pet?<br>o Following recommendations provided by a vet<br>o According to specifications on food packaging<br>o Dog fed until it stops eating<br>o A constant food supply is made available<br>o Other	
**44**	Feeding schedule<br>o Fixed (at the same time every day)<br>o Random<br>o Other	
**45**	Do you give your pet food supplements?<br>o Yes<br>o No	
**46**	What type of supplement (vitamins, fatty acids, etc.)	
**47**	Do you give your pet food rewards?<br>o Yes<br>o No	
**48**	Physical activity<br>o None<br>o Walking	

<table>
<tr><td></td><td>o Running</td><td></td></tr>
<tr><td>49</td><td>Daily exercise (time)
o None
o < 30 min
o 30 min - 1 hour
o > 1 hour
o Other:</td><td></td></tr>
<tr><td>50</td><td>Does your pet suffer from any disease(s)?
o No
o Yes</td><td></td></tr>
<tr><td>51</td><td>What disease?</td><td></td></tr>
<tr><td>52</td><td>What treatment(s) does your pet receive?</td><td></td></tr>
<tr><td>53</td><td>How many times have you had to take your pet to the vet due to health problems during the last year?</td><td></td></tr>
<tr><td>54</td><td>My pet gets sick easily?<table><tr><td>1</td><td>2</td><td>3</td><td>4</td><td>5</td></tr><tr><td>Total disagreement</td><td></td><td></td><td></td><td>Total agreement</td></tr></table></td><td></td></tr>
<tr><td>55</td><td>Do you believe your pet is happy?<table><tr><td>1</td><td>2</td><td>3</td><td>4</td><td>5</td></tr><tr><td>Not happy</td><td></td><td></td><td></td><td>Happy</td></tr></table></td><td></td></tr>
<tr><td></td><td>Owner-pet relationship</td><td></td></tr>
<tr><td>56</td><td>Who is responsible for caring for the animal?
o The person filling out this questionnaire
o Another member of the household
o Several people
o Other</td><td></td></tr>
<tr><td>57</td><td>How many other animals live with the pet?
o None
o 1
o 2
o 3
o 4
o 5
o > 5</td><td></td></tr>
<tr><td>58</td><td>How long do you spend with your pet each day?
You should indicate the active time. Excluding the hours of sleep.
o < 2 hours
o 2 - 4 hours
o 4 - 6 hours
o 6 - 8 hours
o > 8 hours</td><td></td></tr>
<tr><td>59</td><td>Do you share food with your pet while eating?<table><tr><td>1</td><td>2</td><td>3</td><td>4</td><td>5</td></tr><tr><td>Never</td><td></td><td></td><td></td><td>Always</td></tr></table></td><td></td></tr>
<tr><td>60</td><td>Are you with your animal while he/she is eating?</td><td></td></tr>
</table>

<table>
<tr><td></td><td><table><tr><td>1</td><td>2</td><td>3</td><td>4</td><td>5</td></tr><tr><td>Never</td><td></td><td></td><td></td><td>Always</td></tr></table></td><td></td></tr>
<tr><td>61</td><td>Do you think that giving treats or gifts to your dog makes him happier?<table><tr><td>1</td><td>2</td><td>3</td><td>4</td><td>5</td></tr><tr><td>Never</td><td></td><td></td><td></td><td>Always</td></tr></table></td><td></td></tr>
<tr><td>62</td><td>Do you usually sleep with your pet?<table><tr><td>1</td><td>2</td><td>3</td><td>4</td><td>5</td></tr><tr><td>Never</td><td></td><td></td><td></td><td>Always</td></tr></table></td><td></td></tr>
<tr><td>63</td><td>Do you consider your pet a member of the family?
o Yes
o No</td><td></td></tr>
<tr><td>64</td><td>Does taking care of a pet have an influence on your physical activity?
o Yes, I do more exercise
o Yes, I do less exercise
o It does not influence the amount of physical activity I do</td><td></td></tr>
<tr><td>65</td><td>Do you regularly exercise with your pet?
o No
o Always
o Sometimes
o Occasionally
o Hardly ever</td><td></td></tr>
<tr><td>66</td><td>What benefits do you attribute to having a pet?</td><td></td></tr>
<tr><td>67</td><td>What (if any) problems does your pet cause?</td><td></td></tr>
<tr><td></td><td>Obesity background</td><td></td></tr>
<tr><td>68</td><td>Do you consider obesity in people a disease?
o Yes
o No
o Other</td><td></td></tr>
<tr><td>69</td><td>Do you consider obesity in animals a disease?
o Yes
o No
o Other</td><td></td></tr>
<tr><td>70</td><td>Obesity is increasing in society (among both people and pets). What do you think are the three main reasons?
1….
2….
3….</td><td></td></tr>
<tr><td>71</td><td>What would you recommend to stop this increase?</td><td></td></tr>
<tr><td>72</td><td>Do you think that collaboration between healthcare services and vets could be important for combating obesity? Why?</td><td></td></tr>
<tr><td></td><td>Satisfaction</td><td></td></tr>
<tr><td>73</td><td>Difficulty of the survey<table><tr><td>1</td><td>2</td><td>3</td><td>4</td><td>5</td></tr></table></td><td></td></tr>
</table>

	Easy | | | | Tricky	
**74**	Length<br>1 | 2 | 3 | 4 | 5<br>Short | | | | Long	
**75**	Have you found the questionnaire interesting?<br>1 | 2 | 3 | 4 | 5<br>No | | | | Yes	
	**Thank you very much for your help!**	

Supplementary Information 2. Detailed descriptive data of the participants and their dogs in each country.

	Croatia	Denmark	Italy	Lithuania	Poland	Portugal	Romania	Serbia	Spain	Sweden
**Total number of responces obtained**	**233**	**541**	**126**	**260**	**406**	**298**	**218**	**160**	**208**	**735**
**The questionnaire dissemination ways (%)**										
Through a researcher involved in the study	13,7	2,8	11,1	37,7	28,1	40,3	10,7	23,8	18,8	0,4
Through the veterinary clinic I go to with my pet	18,0	7,4	37,3	1,5	19,5	1,0	4,6	1,9	31,3	0,1
Through somebody else who went to a veterinary clinic	3,9	0,7	0,8	1,2	2,0	0,0	0,9	0,0	1,4	0,0
Through a dog activity or dog breeding organisation	1,3	1,1	0,0	0,0	0,0	0,7	0,6	0,0	0,5	0,3
Through a colleague	26,6	1,8	28,6	10,0	7,9	11,4	4,6	0,2	8,2	2,4
Through a friend or family member	15,5	4,8	21,4	7,3	9,6	14,4	3,2	26,3	21,2	2,9
Through social media (e.g. Facebook, Twitter)	19,3	80,8	0,8	36,2	30,0	32,2	74,8	28,1	18,8	93,7
Other	1,7	0,6	0,0	6,2	3,0	0,0	0,5	0,0	0,0	0,1
**Owner Data**										
**Man/Woman**	59 (25,3%)/ 174 (74,7%)	49 (9,1%)/ 492 (90,9%)	35 (28%)/ 91 (72%)	48 (18,5%)/212 (81,5%)	67 (16,5%)/ 339 (83,5%)	50 (16,8%)/248 (83,2%)	39 (17,9%)/179 (82,1%)	42 (26,3%)/ 118 (73,7%)	58 (27,9%)/ 150 (72,1%)	38 (5,2%)/697 (94,8%)
**Age, years**	18 - 73 (n=233)	18 - 84 (n=541)	19 - 80 (n=126)	18 - 80 (n=260)	18 - 72 (n=406)	18 - 72 (n=298)	18 - 68 (n=218)	18 - 72 (n=160)	18 - 63 (n=208)	18 - 98 (n=735)
**Number of family members living with pet**	3 (1-7)	2 (1-7)	3 (1 - 6)	3 (1-8)	3 (1 - 8)	3 (1 - 8)	3 (1-10)	3 (1 - 7)	3 (1-6)	2 (1-7)
**Educational level of person responding to questionnaire**										
o Primary	0	30 (5,5%)	0	1 (0,4%)	5 (1,2%)	2 (0,7%)	26 (11,9%)	0	7 (3,4%)	25 (3,4%)
o Secondary/High School	77 (33,0%)	45 (8,3%)	29 (23,0%)	71 (27,3%)	160 (39,4%)	45 (15,1%)	2 (0,9%)	38 (23,8%)	11 (5,3%)	191 (36,0%)
o Vocational training	11 (4,7%)	184 (34,0%)	17 (13,5%)	14 (5,4%)	16 (3,9%)	2 (0,7%)	1 (0,5%)	6 (3,8%)	60 (28,8%)	102 (13,9%)
o University degree	108 (46,4%)	169 (31,2%)	65 (51,6%)	135 (51,9%)	212 (52,2%)	185 (62,1%)	132 (60,6%)	80 (50,0%)	94 (45,2%)	403 (54,8%)
o Postgraduate qualifications	35 (15,0%)	58 (10,7%)	13 (10,3%)	34 (13,1%)	12 (3,0%)	63 (21,1%)	57 (26,1%)	36 (22,5%)	35 (23,3%)	13 (1,8%)
o Other:	2 (0,9%)	55 (10,2%)	2 (1,6%)	6 (2,3%)	1 (0,2%)	1 (0,3%)	-	0	1 (0,5%)	1 (0,1%)
**Employment ***										
o Student	22 (9,4%)	74 (13,7%)	8 (6,3%)	109 (41,9%)	181 (44,6%)	104 (34,9%)	51 (23,4%)	29 (18,1%)	14 (9,3%)	120 (16,3%)
o Employed	177 (76,0%)	364 (67,3%)	100 (79,4	131 (50,4%)	187 (46,1%)	171 (57,4%)	147 (67,4%)	110 (68,8%)	162 (77,9%)	480 (65,3%)
o Retired	13 (5,6%)	62 (11,5%)	10 (7,9%)	5 (1,9%)	9 (2,2%)	8 (2,7%)	11 (50,5%)	7 (4,4%)	0	71 (9,7%)
o Unemployed	16 (6,9%)	41 (7,6%)	8 (6,3%)	4 (1,5%)	28 (6,9%)	15 (5,0%)	9 (4,1%)	14 (8,8%)	32 (15,4%)	29 (3,9%)
o Other:	5 (2,1%)	0	0	11 (4,2%)	0	0	0	0	0	35 (4,8%)
**Monthly Family income ***										
o =< national minimum wage (MW)	39 (16,7%)	32 (5,9%)	17 (13,5%)	21 (8,1%)	83 (20,4%)	79 (26,5%)	5 (2,3%)	35 (21,9%)	28 (13,5%)	115 (15,6%)
o 1 – 2 MW	0	81 (15,0%)	46 (36,5%)	61 (23,5%)	91 (22,4%)	108 (36,2%)	25 (11,5%)	53 (33,1%)	54 (26,0%)	222 (30,2%)
o 2 – 3 MW	94 (40,3%)	98 (18,1%)	36 (28,6%)	58 (22,3%)	86 (21,2%)	72 (24,2%)	2 (0,9%)	47 (29,4%)	49 (23,6%)	219 (29,8%)
o 3 – 4 MW	82 (35,2%)	202 (37,3%)	8 (6,3%)	49 (18,8%)	75 (18,5%)	1 (0,3%)	109 (50,0%)	16 (10,0%)	32 (15,4%)	107 (14,6%)
o => 4 MW	18 (7,7%)	128 (23,7%)	19 (15,1%)	71 (27,3%)	71 (17,5%)	38 (12,8%)	77 (35,3%)	9 (5,6%)	45 (21,6%)	72 (9,8%)
**During the past 30 days (1 month) on how many days did you smoke?**										
o Every day or almost every day	76 (32,6%)	96 (17,7%)	31 (24,6%)	50 (19,2%)	95 (23,4%)	49 (16,4%)	78 (35,8%)	50 (31,3%)	29 (13,9%)	98 (13,3%)
o Some days	10 (4,3%)	19 (3,5%)	12 (9,5%)	20 (7,7%)	9 (2,2%)	25 (8,4%)	10 (4,6%)	10 (6,3%)	14 (6,7%)	30 (4,1%)
o Not smoker	147 (63,1%)	426 (78,7%)	83 (65,9%)	190 (73,1%)	302 (74,4%)	224 (75,2%)	130 (59,6%)	100 (62,5%)	165 (79,3%)	607 (82,6%)
**Disease, Yes/No**	64 (27,5)/ 169 (72,5%)	202 (37,3%)/ 339 (62,7%)	32 (25,4%)/ 94 (74,6%)	56 (21,5%)/204 (78,5%)	110 (27,1%)/ 296 (72,9%)	74 (24,8%)/ 224 (75,2%)	44 (20,2%)/ 174 (79,8%)	30 (18,8%)/ 130 (81,2%)	49 (23,6%)/ 159 (76,4%)	218 (29,7%)/517(70,3%)
**Treatment, Yes/No**	56 (24,0%)/ 177 (76,0%)	167 (30,9%)/ 374 (69,1%)	35 (27,8%)/ 91 (72,2%)	46 (17,7%)/214 (82,3%)	40 (10,0%)/ 366 (60,0%)	61 (20,5%)/ 237 (79,5%)	28 (12,8%)/190 (87,2%)	26 (16,3%)/ 134 (83,7%)	39 (18,8%)/ 169 (81,2%)	176 (23,9%)/559 (76,1%)
**Dog Data**										
**Age, years**	0,0 - 15,0 (n=233)	0,0 - 22,0 (n=539)	0,3 - 16,0 (n=126)	0,1 - 15,0 (n=260)	0,3 - 16,0 (n=406)	0,0 - 22,0 (n=298)	0,3 - 16 (n=216)	0,1 - 17,0 (n=160)	0,1 - 16,0 (n=208)	0,1 - 15
**Sex, Female/Male**	124 (53,2%)/ 109 (46,8%)	266 (49,2%)/ 275 (50,8%)	65 (51,6%)/ 61 (48,4%)	128 (49,2%)/132 (50,8%)	224 (55,2%)/ 182 (44,8%)	148 (49,7%)/ 150 (50,3%)	85 (39,0%)/ 133 (61,0%)	81 (50,6%)/ 79 (49,4%)	114 (54,8%)/ 94 (45,2%)	383 (52.1%)/352 (47,9%)
**Breed** (5 breeds most repeated, with the exception of mixed breed/mongrel dogs, that were the most popular in all the studied countries)	Maltese (n=40), German Shepherd (n=8), Staffordshire Bull Terrier (n=8), Golden Retriever (n=7), Labrador Retriever (n=7)	Labrador Retriever (n=65), Golden Retriever (n=34), German shepherd (n=23), Border Collie (n=21), Poodle (n=17)	German Shepherd (n=7), Labrador Retriever (n=6), Pinscher (n=5), Cocker Spaniel (n=4), Border Collie (n=3), French Bulldog (n=3), Doberman (n=3), Jack Russell	German shepherd (n=13), Yorkshire terrier (n=10), Belgian Shepherd (n=8), Labrador Retriever (n=8), and Dachshund (n=8)	German Shepherd (n=24), Labrador Retriever (n=19), Schnauzer (n=16), Briard (n=12), Golden Retriever (n=8)	Labrador Retriever (n=32), German shepherd (n=10), Golden Retriever (n=8), Jack Russell Terrier (n=8), Yorkshire terrier (n=7)	Labrador Retriever (n=13), German shepherd (n=11), Golden Retriever (n=10), Yorkshire terrier (n=7), Siberian Husky (n=5)	Labrador Retriever (n=12), Yorkshire Terrier (n=6), Maltese (n=6), Rottweiler (n=6), Golden Retriever (n=6)	Labrador Retriever (n=10), Golden Retriever (n=7), Bodegero andaluz (n=7), Boxer (n=6), Podenco (n=6)	Labrador Retrieves (n=79), Pug (n=40), Deutscher Schäferhund (29), Border Collie (n=27), Golden retriever (n=21).
**Reproductive status:**										
o Intact	131 (56,2%)	329 (60,8%)	61 (48,4%)	181 (69,6%)	197 (48,5%)	155 (52,0%)	123 (56,4%)	108 (67,5%)	75 (36,1%)	506 (68,8%)
o Neutered at <6 months	17 (7,3%)	16 (3,0%)	11 (8,7%)	6 (2,3%)	19 (4,7%)	22 (7,4%)	11 (5,0%)	4 (2,5%)	17 (8,2%)	7 (1,0%)
o Neutered at 6-11 months	20 (8,6%)	18 (3,3%)	24 (19,0%)	23 (8,8%)	34 (8,4%)	36 (12,1%)	15 (6,9%)	6 (3,8%)	36 (17,3%)	54 (7,3%)
o Neutered at 1 year	10 (4,3%)	17 (3,1%)	7 (5,6%)	7 (2,7%)	27 (6,7%)	13 (4,4%)	9 (4,1%)	5 (3,1%)	0	0 (0%)
o Neutered at 1-2 years	12 (5,2%)	30 (5,5%)	9 (7,1%)	11 (4,2%)	46 (11,3%)	16 (5,4%)	16 (7,3%)	12 (7,5%)	32 (15,4%)	70 (9,5%)
o Neutered at 2-8 years	32 (13,7%)	102 (18,9%)	8 (6,3%)	24 (9,2%)	64 (15,8%)	36 (12,1%)	36 (16,5%)	18 (11,3%)	36 (17,3%)	85 (11,6%)
o Neutered at >8 years	11 (4,7%)	27 (5,0%)	6 (4,8%)	5 (1,9%)	16 (3,9%)	14 (4,7%)	4 (1,8%)	4 (2,5%)	11 (5,3%)	11 (1,5%)
o Not sure	0	2 (0,4%)	0	3 (1,2%)	3 (0,7%)	6 (2,0%)	4 (1,8%)	3 (1,9%)	1 (0,5%)	2 (0,3%)
**Disease, Yes/No**	28 (12,0%)/ 205 (88,0%)	122 (22,6%)/ 419 (77,4%)	43 (34,1%)/ 83 (65,9%)	47 (18,1%)/ 213 (81,9%)	97 (23,9%)/ 309 (76,1%)	53 (17,8%)/ 245 (82,2%)	26 (11,9%)/ 192 (88,1%)	25 (15,6%)/ 135 (84,4%)	50 (24,0%)/ 158 (76%)	126 (17.1%)/609 (82,9%)
**Treatment, Yes/No**	19 (8,2%)/ 214 (91,8%)	94 (17,4%)/ 447 (82,6%)	34 (27,0%)/ 92 (73,0%)	29 (11,2%)/ 231 (88,8%)	85 (20,9%)/ 321 (79,1%)	48 (16,1%)/ 250 (83,9%)	21 (9,6%)/ 197 (90,4%)	21 (13,1%)/ 139 (86,9%)	43 (20,7%)/ 165 (79,3%)	116 (15,8%)/619 (84,2)
**Visits to vet during last year, because of health problems**	0-10	0-15	0 - 24	0 - 10	0-50	0-48	0-48	0 - 24	0-14	0 - 25

Suplementary Information 3. Prevalence of obesity based on BCS categories cross tabulated with BFI categories [Total (Percent)].

**BCS categories**	**BFI categories**		**Total (percent)**
	**Normal weight,** BFI 20%	**Overweight,** BFI 30-70%	
**Underweight**, BCS 1-2	N = 369 (11.8%)	N = 84 (2.7%)	453 (14.5%)
**Normal weight**, BCS 3	N = 1139 (36.5%)	N = 939 (30.1%)	2078 (66.6%)
**Overweight**, BCS 4-5	N = 32 (1.1%)	N = 556 (17.8%)	588(18.9%)
**Total**	N = 1540 (49.9%)	N = 1579 (50.6%)	3119 (100.0%)

Chi-squared test, $\chi 2(2)$ = 665,14, p< 0,001
